# Factors influencing the success of aerial rabies vaccination of foxes

**DOI:** 10.1038/s41598-017-14615-2

**Published:** 2017-10-30

**Authors:** J. Henning, E. Giorgi, R. J. Soares Magalhães, P. Tizzani, P. Viviani, N. Pejovic, M. Hrapović, C. Potzsch

**Affiliations:** 1 School of Veterinary Science, The University of Queensland, Gatton, 4343 Qld Australia; 2 Faculty of Health and Medicine, Furness College, Lancaster University, Lancaster, LA1 4YW United Kingdom; 3 Children’s Health and Environment Program, Child Health Research Centre, The University of Queensland, South Brisbane, 4101 Qld Australia; 4 Department of Veterinary Sciences, University of Turin, Grugliasco (Torino), 10095 Italy; 5 Veterinary consultant, Perugia, 06123 Italy; 6 Diagnostic Veterinary Laboratory, Podgorica, 81000 Montenegro; 7 Veterinary Administration of the Republic of Montenegro, Podgorica, 81000 Montenegro; 8 Veterinary epidemiology consultant, Tramnitz, 16866 Germany

## Abstract

Sylvatic rabies has been eradicated from most of Central Europe, but cases still occur in the Balkans. Oral rabies vaccination of foxes is an effective method for controlling the disease. The aim of this study was to evaluate the success of aerial vaccination campaigns conducted in Montenegro by identifying ecological, environmental and climatic factors that influenced the prevalence of antibodies to the rabies vaccine. To monitor the bait uptake and the serological responses to vaccination, foxes were shot by hunters. Of 175 shot foxes, 142 foxes (81.1%) had consumed baits. Of these only a total of 81 (57.0%) tested positive for rabies vaccine antibodies, possibly, due to the delayed uptake of bait in which the rabies vaccine was already inactivated. We found that low vaccination responses were associated with high fox density and bait delivery in open areas. In high fox density habitat, bait uptake might be delayed as other food and prey options for foxes are abundant. Similarly, delayed bait uptake probably occurred in open areas as such areas are less frequently used by foxes. The findings of this study suggest that efficacy of oral rabies vaccination by aerial delivery is associated with landscape features.

## Introduction

Oral rabies vaccination of foxes is known to be an effective method for controlling rabies in this species^1^. Delivery of baits is conducted using airplanes and baits are distributed along parallel flight lines. A distribution by hand is the preferred methods in suburban areas^2^. Usually, industrially manufactured baits are used – they are made of an envelope attractive for foxes containing a capsule or a plastic sachet filled, most commonly, with an attenuated anti-rabies vaccine in liquid form^2^.

Although rabies had been eradicated from most of Europe by 2010, there were still cases occurring in Eastern Europe and in the Balkan region^3–5^. Rabies was endemic in Montenegro until the start of oral vaccination in 2010, with a total of 43 (of 69), 49 (of 113) and of 69 (of 119) animals tested positive in Montenegro in 2008, 2009 and 2010, respectively (http://www.who-rabies-bulletin.org/Queries/Surveillance.aspx)^6^. In 2010, 55 (79%) of the animals that tested positive were foxes. The number of animals tested for rabies represents animals indicative of rabies symptoms or rabies suspicious animals to which humans were exposed.

To combat sylvatic rabies in the Balkan, a number of EU funded projects were established in the region. In Montenegro, an EU funded rabies control project was carried out between 2010 and 2013 (EuropeAid/128207/C/SER/ME) with the aim to design and implement an oral rabies vaccination campaign, to improve diagnostic, monitoring and control capacities of veterinary services and to align legal and regulatory framework for rabies control with the EU Guidelines. The EU funded the first two oral vaccination campaigns using aerial baiting of foxes in autumn 2011 and spring 2012 while later campaigns were conducted by the Montenegrin government.

Despite the overwhelming success of aerial vaccinations, clusters of rabies cases might still remain and require targeted actions^7^. Previous research highlighted that the success of oral rabies vaccination programs depended on bait delivery methods, the proportion of land area affected by rabies as well as the size and overlap of successive vaccinations^8,9^. The holistic identification of the impact of environmental, climatic, wildlife density and vaccination campaign-associated (e.g. bait density) risk factors on rabies vaccination prevalence could help policy makers to implement specific actions to improve the efficacy of rabies control programs.

In this study we aimed to identify ecological, environmental and climatic factors that influenced rabies vaccination antibody prevalence, quantify the role of risk factors in the spatial variation of rabies vaccination antibody prevalence and generate a predictive map of the prevalence of antibodies to rabies vaccination.

## Methods

### Vaccination campaigns

Two nationwide aerial rabies vaccination programs were conducted in autumn 2011 (3–11 October 2011) and in spring 2012 (27 April –2 May 2012). A total of five airplanes were deployed for each bait delivery program and the aerial distribution was completed over a period of nine and six days, respectively. Each of the planes was equipped with a GPS device to georeference the location of the bait release. It was aimed to deliver the bait in a density of 20 baits per square kilometre at a flight lines distance of 500 m. Density of 20 baits per square kilometre are considered as adequate for aerial vaccination programs of foxes^2^. Each bait contained one virus vaccination dose of the attenuated *LYSVULPEN* rabies vaccine (Bioveta, Czech Republic) encapsulated in an aluminium plastic blister. The blister was embedded in a feed mixture attractive for foxes and contained tetracycline HCl as an indicator for bait ingestion.

### Bait consumption and rabies vaccination antibodies

To monitor the bait uptake and the serological responses following vaccination, foxes were shot by hunters. The shooting of foxes is part of national regulations that need to be implemented to evaluate the effectiveness of rabies vaccination campaigns^6^. Thus foxes were shot by hunters in Montenegro as part of their duties as stated in Article 5.7 of the “Regulation about measures for detection, control and eradication of rabies in animals” (Official Journal of the Republic of Montenegro No. 17/2007, 31.12.2007). The lower jaw of foxes was removed by hunters and submitted to the Diagnostic Veterinary Laboratory (DVL) of Montenegro to detect tetracycline as indicator for bait uptake (using florescence methods) and to determine the age of the foxes from the teeth. From each shot fox a blood sample or abdominal fluid was collected and submitted to DVL for detecting rabies vaccination antibodies using the BioPro blocking ELISA (O.K. Servis, BioPro, Czech Republic). The OD value was read at 450 nm and the ELISA percentage of blocking or inhibition was calculated. This measure was used to classify samples as either positive or negative for rabies vaccine antibodies (http://www.rabieselisa.com/resultsinterpretation/).

### Observed rabies vaccination antibody prevalence

The location where foxes were shot was identified by hunters on topographical maps and recorded into sample submission forms sent to DVL. A 1 km × 1 km vector grid was developed and overlaid over a digital map of Montenegro. As fox home territories range between 40 and 400 ha^2,10^ an one square kilometre grid cell was used to represent a fox territory of 100 ha. Coordinates of shot fox locations were plotted within the 1 km vector grid and observed rabies antibody prevalence was calculated for each grid cell as the *Number of foxes that consumed bait and tested positive* *per grid cell* over the *Total number of foxes that consumed bait per grid cell*.

### Risk factor data

Different types of variables were considered as risk factors that could influence rabies vaccination antibody prevalence, including bait density, wildlife density, environmental and climate variables (Table 1). The same 1 km vector grid used to summarize the rabies antibody prevalence was utilized for generating the risk factor data.

**Table 1:** Risk factor data analysed for association with rabies antibody prevalence following an aerial vaccination program of foxes in Montenegro, 2011–2012.

**Bait density data**
Bait density in 2011 (baits/km^2^)
Bait density in 2012 (baits/km^2^)
**Wildlife density data**
Mean fox density (animals/km^2^)
Mean total carnivore density (animals/km^2^
**Environmental variables**
Altitude (meters)
Mean Normalized Difference Vegetation Index (NDVI; ranging from −1 to + 1)
Mean distance from agricultural areas (metres)
Mean distance from urban areas (metres)
Mean distance from forest areas (metres)
Mean distance from water areas (metres)
Mean distance from wetland areas (metres)
Mean distance from urban fabrics (metres)
Mean distance from industrial complexes (metres)
Mean distance from mines and dumps (metres)
Mean distance from artificial, non-agricultural vegetated areas (metres)
Mean distance from arable land (metres)
Mean distance from permanent crops (metres)
Mean distance from pastures (metres)
Mean distance from heterogeneous agricultural areas (metres)
Mean distance from forests (metres)
Mean distance from scrub and/or herbaceous vegetation associations (metres)
Mean distance from open spaces with little or no vegetation (metres)
Mean distance from inland wetlands (metres)
Mean distance from maritime wetlands (metres)
Mean distance from marine waters (metres)
Percentage of pasture areas per km^2^
Percentage of complex cultivation patterns areas per km^2^
Percentage of land area principally occupied by agriculture with natural vegetation per km^2^
Percentage of broad-leaved forest areas per km^2^
Percentage of coniferous forest areas per km^2^
Percentage of mixed forest areas per km^2^
Percentage of natural grasslands areas per km^2^
Percentage of transitional woodland-shrub areas per km^2^
Percentage of sparsely vegetated areas per km^2^
Percentage of inland marshes areas per km^2^
**Climate variables**
Mean temperature until 2 weeks after the end of the campaign (in Celsius)
Number of days with snow cover until 2 weeks after the end of the campaign

Bait density was estimated as described in Tizzani *et al*., 2013^11^ representing the total number of baits in each 1 km × 1 km grid square. A total of 274,219 baits were distributed during the 2011 and 273,888 baits in the 2012 vaccination campaign, with a mean bait density of 19.9 baits/km^2^ in 2011 and 20.0 baits/km^2^ in 2012. Approximately 57% of land mass of Montenegro had a bait density ≥20 baits/km^2^.

Wildlife density (fox and total carnivore density) analysis was conducted as described in Tizzani and Sasic, 2013^12^. Indirect faecal counts were conducted in 73 transects spanning a length 834 km and direct night counts were conducted over 43 transects totalling a length of 503 km and wildlife density was calculated for each transect. The mean fox density was 3.9 foxes/km^2^ for faecal counts and 3.6 foxes/km^2^ for night counts. Then wildlife density was calculated for un-sampled grid cells by spatial interpolation using ordinary kriging^12^.

Climate variables were derived from MODIS satellite images (https://modis.gsfc.nasa.gov/about/). These included hdf raster files of daily snow cover data and daily land surface temperature data from the beginning of the vaccination campaign until 14 days after the last day of bait delivery in 2011 and 2012. Values from these raster images were extracted for each grid cell using the Spatial Analysist tool in ArcGIS. Land surface temperature was averaged and days with snow cover were summed for each grid cell for each vaccination campaign.

The NDVI values were derived from the Glovis USGS website (https://glovis.usgs.gov/) using the Landsat scenes for 2012. NDVI values were calculated for each scene using the near infrared and red wave lengths. Usually Landsat scenes are available in 16 days cycles, but some were discarded due to cloud cover. After the calculation of all the NDVI values for one year, the average NDVI values for each grid cell were produced using a raster calculator.

Environmental raw data (altitude and percentage land cover variables) were obtained or calculated (mean distance variables) from values stored in the CORINE LAND COVER 2006 database (http://www.eea.europa.eu/publications/COR0-landcover).

### Data analysis

A binominal Generalised Linear Model (GLM) with a logit link function was used to model the association between rabies vaccination antibody prevalence for 2011 and 2012 and potential risk factors. To assess the relative importance of each risk factor, the explanatory variables in the model were standardized to have a mean of zero and a standard deviation of one. Correlation between risk factors were assessed by developing a matrix plot and by calculating pairwise Pearson correlations. If high bivariate correlations (Pearson correlation coefficient >0.7) were present, one of the two variables was not included in the further analysis. Risk factors with a p-value < 0.2 in the univariate analysis were included in the multivariable model. A forward stepwise algorithm was used to carry out variable selection in the multivariable model^13^. Likelihoods of the full and the reduced models were compared using the log-likelihood ratio (LR) test^13^. We also evaluated if adding or removing a variable would result in at least a 20% relative change in the effects of any of the remaining variables and, if this was the case, we then kept or, if not present, included such a variable in the regression^14^.

Analysis of the residuals was conducted by visually assessing any potential lack of fit of the model and by using the Pearson’s Chi Square test to validate whether or not there was sufficient evidence that the observed data did not fit the model. A non-significant Pearson’s Chi Square test indicates that there is no reason to assume that the model is not correct. Coefficients were presented as odds ratios with 95% confidence intervals^13^. We also reported the ROC area under the curve for the fitted models in 2011 and 2012 using the full data-sets.

To quantify the spatial autocorrelation of the rabies vaccination antibody prevalence, semivariograms were produced for each vaccination campaign, i.e. for the fox locations and the observed rabies vaccination antibody prevalence for 2011 and 2012. After adjusting for the risk factors in our final multivariable model we tested for the presence of residual spatial autocorrelation by using a Monte Carlo approach based on the empirical semivariogram, whose full description is available in the in the ‘Supplementary Information’. This algorithm generates 95% confidence intervals for the empirical semivariogram, at separating distances between pairs of points, under the hypothesis of spatial independence of the logistic regression Pearson’s residuals. If the observed empirical semivariogram from the fitted model falls within the 95% tolerance bandwidth, we then conclude that the data do not show evidence of residual spatial autocorrelation.

We also explored the impact of home ranges larger than 1 square kilometre and up to 4 square kilometres, on the estimates of the regression coefficients for the different risk factors included in the final model. To this end, we modelled the location where the fox consumed the bait as an inhomogeneous Poisson process with intensity function given by the estimated fox density. Since each grid cell corresponded to a 1 square kilometre home rage area, we then considered, for a given home range of H square kilometres, all possible combinations of grid cells adjacent to that where the fox was shot, such that their total area is H-1. The resulting likelihood function from a single observation is then a weighted average of binomial likelihood functions with weights proportional to the fox density attached to each of the adjacent grid cells. In this way we were able to account for the spatial uncertainty in each of the environmental variables by letting these vary according to their observed values in the adjacent grid cells. We provide detailed information on this modelling approach in the ‘Supplementary Information’.

STATA SE 14 (StataCorp, Lakeway Drive, College Station, Texas 77845 USA) was used for model fitting and residual analysis and R 3.3.2. (2016 The R Foundation for Statistical Computing) was used to carry out the test for spatial correlation, generate the predictions for each grid cell centroid (predicted prevalence and standard error of prediction, using bootstrapping to estimate the latter) and to assess the impact of larger home ranges on the model coefficients.

### Mapping

ArcMap 10.4 (1999–2005 Esri Inc.) was used to produce a point location map of observed rabies vaccination antibody prevalence per centroid grid cell and raster maps of predicted rabies antibody vaccination prevalence and their standard errors for 2001 and 2012. The projected coordinate system MGI Balkan zone 7 was used for mapping. The raster maps of the predicted prevalence and the standard error were calculated based on each centroid using the inverse distance weighted (IDW) technique, thus interpolating the centroid data into a raster surface, in the ArcGIS Spatial Analyst.

## Results

### Observed rabies vaccination antibody prevalence

A total of 252 shot fox locations were recorded. A total of 35 locations had incorrect coordinates (i.e. coordinates were within water bodies or outside of the boundaries of Montenegro) and were excluded from the analysis. Of the 217 foxes for which correct coordinates were recorded, blood samples had been obtained from 175 foxes. Of these 175 foxes, 142 foxes (81.1%, 95%: CI 75, 96) had positive tetracycline results – hence these foxes had consumed the vaccine baits distributed and were used in the further data analysis.

A total of 81 of the 142 bait-consuming foxes (57.0%, 95% CI: 49, 65) tested positive for rabies vaccine antibodies over the observation period of two years. In 2011, of the 86 foxes that consumed bait, 58.1% (N = 50) tested positive, where in 2012 of the 56 foxes that consumed bait, 55.4% (N = 31) tested positive.

The number of grid cells (spatial resolution of 1 km × 1 km) with locations of bait-consuming foxes was 76 for 2011 and 50 for 2012.

### Risk factors for rabies vaccination antibody titres

Covariates or risk factor variables were extracted for a total of 12,797 grid cells covering the territory of Montenegro (excluding water and urban areas). In 2011, 76 grid cells with fox locations and in 2012 50 grid cells with fox locations were used for risk factor analysis. The final multivariable models of risk factors associated with rabies vaccination antibody prevalence are listed in the tables below (Table 2).

**Table 2:** Multivariable model of risk factors associated with rabies vaccination antibody prevalence of foxes in Montenegro in 2011 and 2012.

Year	Risk factors	Adjusted OR	95% CI	p-value
2011	Standardized mean fox density (animals/km^2^)	0.59	0.36; 0.98	0.043
	Standardised mean distance from open spaces with little or no vegetation (metres)	0.51	0.27; 0.97	0.040
	Standardised percentage of natural grasslands areas (per km^2^)	0.59	0.32; 1.10	0.095
	Standardised mean distance from water areas (metres)	1.57	0.88; 2.81	0.124
2012	Standardized mean Normalized Difference Vegetation Index	2.87	1.14; 7.22	0.047

Fox density was associated with the development of vaccination titres in the 2011 vaccination campaign. In 2011, for an increase by one fox in fox density per square kilometre, there was a 70% increase in the odds of foxes being seronegative (1/0.59 for Standardized mean fox density = 1.69). Also, an increase in standardised mean distance from open spaces with little or no vegetation increased the odds of foxes being seronegative in 2011. The variable ‘Standardised mean distance from water areas’ was not significant at p < 0.05, but removing the variable resulted in a 21% change of ‘Standardised percentage of natural grasslands areas (per km^2^)’ – therefore this variable was retained as potential cofounder in the model for 2011. Similarly, the variable ‘Standardised percentage of natural grasslands areas (per km^2^) was not significant at p < 0.05, but its removal resulted in a 39% change of ‘Standardised mean distance from water areas’ and therefore was kept in the model for 2011. In 2012, increases in the mean NDVI were associated with foxes being more likely positive for rabies vaccine antibodies.

**Figure 1:**
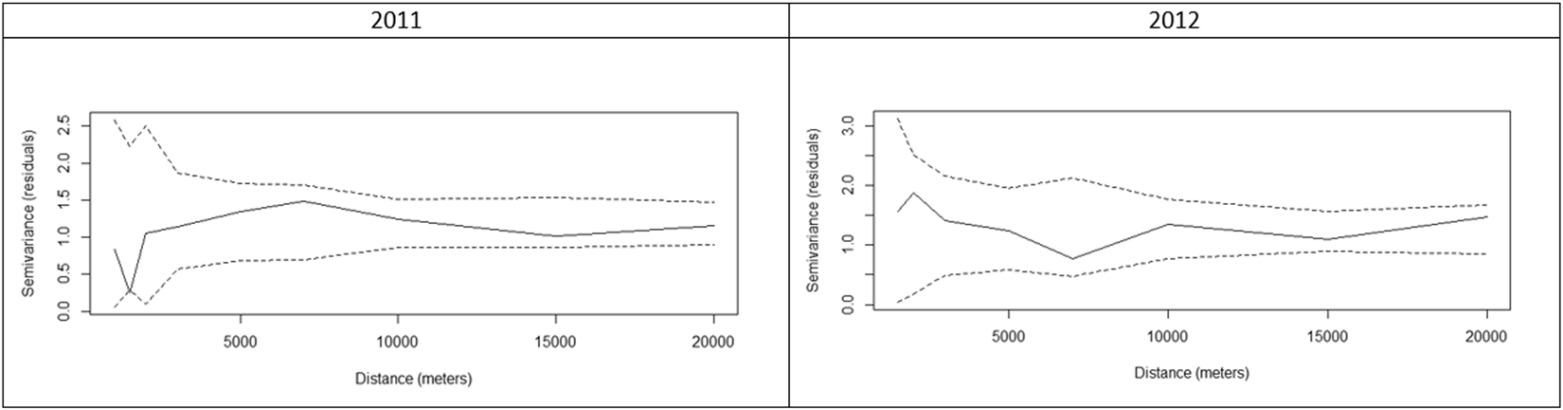
Empirical semivariograms of the Person’s residuals with 95% tolerance interval (generated under the hypothesis of spatial independence) for models of risk factors associated with rabies vaccination antibody prevalence for 2011 and 2012.

The p-values for Pearson Chi Square statistics were 0.266 for 2011 and 0.426 for 2012, therefore the assumption of a Binomial distribution is compatible with the data. Figure 1 shows the semivariogram of the Person’s residuals from the two fitted models. Since these fell within 95% tolerance bandwidth generated under the hypothesis of spatial independence, we concluded that the model residuals (which provided a measure of the unexplained variation in the data) showed no evidence of spatial autocorrelation.

### Model predictions

**Figure 2:**
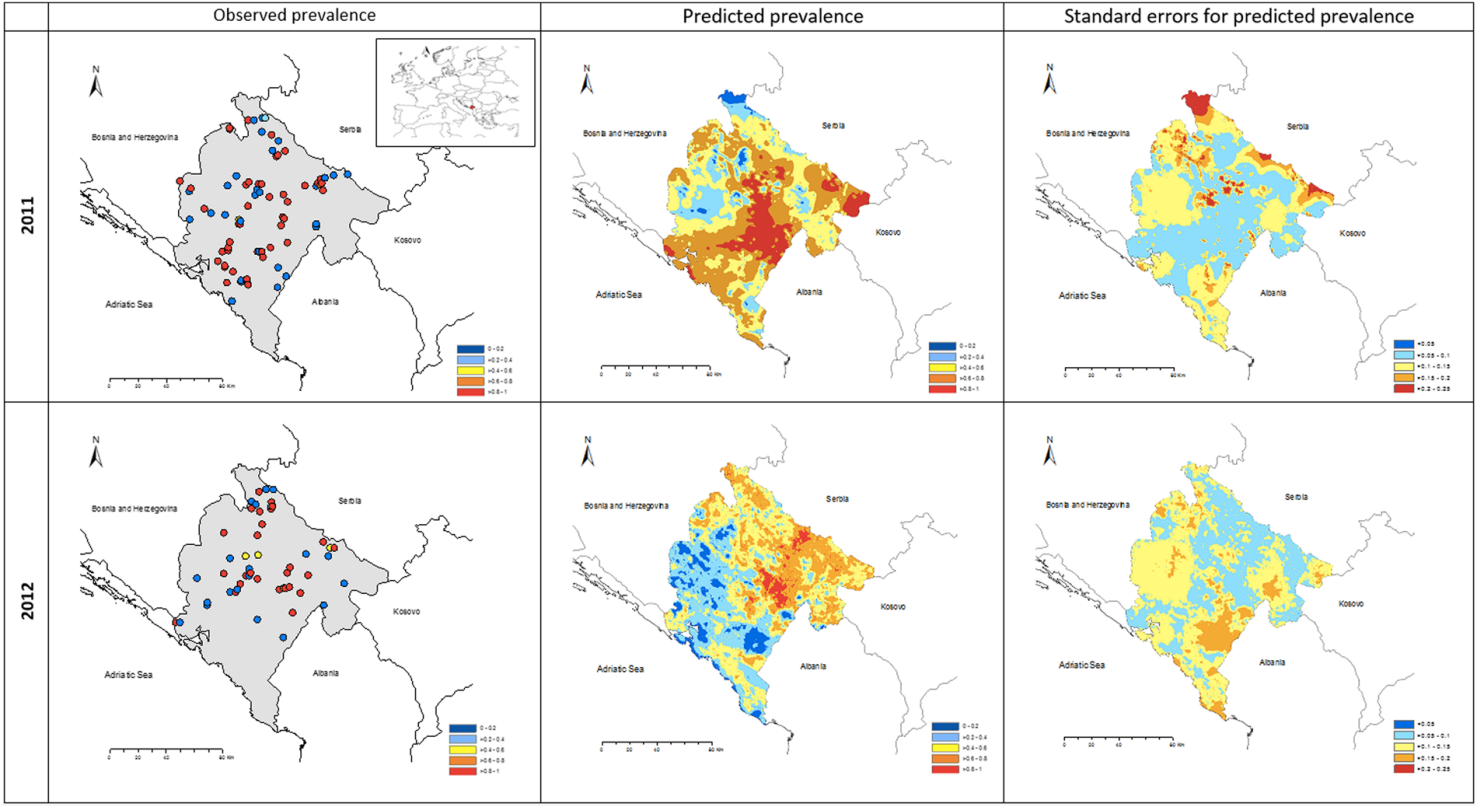
Observed and predicted prevalence and standard errors for predicted prevalence of rabies vaccination antibodies in foxes in Montenegro. Insert shows a map of Europe with Montenegro highlighted in red. Maps were created using ArcMap 10.4 (https://www.arcgis.com) software.

The observed rabies vaccination antibody prevalence and the predicted rabies vaccination antibody prevalence, including its standard errors are shown for both years of the vaccination campaign in Fig. 2. The ROC area under the curve is 0.682 (95% CI: 0.547, 0.816) for 2011, and 0.725 (95% CI: 0.576, 0.874) for 2012.

The predictive prevalence maps demonstrate lower rabies vaccination antibody prevalence in the western (in 2011) and in the south-western part (in 2012) of Montenegro. In 2011 low predicted rabies vaccine prevalence was observed in regions of the country where the fox density was high. Interestingly there were was larger uncertainty in the predicted prevalence in the eastern border region in 2011 and in the southern border region in 2012. In general, larger predicted standard errors were often associated with low predicted prevalence (<0.2).

### Impact of home ranges larger than 1 square kilometre on parameter estimation and model predictions

As expected, larger home ranges lead to a higher uncertainty about the estimated association between the explanatory variables, reported in the previous section, and the probability of presence of rabies antibodies. This is reflected in Figs 2 and 3 of the ‘Supplementary Information’, where the width of the 95% confidence intervals of the regression coefficients increases as home range increases. The variables that are most affected by the variation in home range levels are ‘Percentage of grasslands areas’, for 2011, and NDVI, for 2012, with a 10.7% and a 23.6% relative change in the regression coefficients estimates for a home range of 1 square kilometre with respect to a home range of 4 square kilometres, respectively. However, the effects of home range on the parameter estimates translate into very small effects on the model predictions, as shown by Figs 4 and 5 of ‘Supplementary Information’. Tangible changes are observed only for 2011, with a slight increase in the standard error predictions as home range increases.

## Discussion

Three methods are used to evaluate oral rabies vaccination programs: (1) testing of target species for the occurrence of biomarkers (usually tetracycline) to evaluate the bait uptake (2) examining sera from the target species for rabies virus neutralising antibodies to assess antibody prevalence or/and herd immunity; (3) analysing the incidence of rabies in animals before, during and after the oral vaccination programme^2^. Previous research focussed on either describing bait uptake, the proportion of foxes tested positive or on modeling the bait delivery and the impact of landscape on rabies cases occurring under vaccination programs^8,9^. This is the first detailed analysis of risk factors that impacted on rabies vaccination antibody prevalence following an aerial vaccination program. Our analysis focussed on foxes that consumed bait to identify risk factors that impacted on the development of vaccination titres. We hypothesised that factors influencing the probability of foxes developing rabies vaccine antibodies are related to the viability and thermostability of the vaccine after bait delivery.

Our results indicate that bait uptake in foxes was well above previous studies in Europe and North America in that approximately 81% of the shot foxes were detect positive to tetracycline. Studies in Europe and North America have described that bait uptake can range between 40 and 80%^15^. In a two year vaccination campaign in Kosovo, average bait uptake was 55.7%^16^, while it was 70% in Greece after one vaccination campaign^17^.

Most bait uptake studies have demonstrated that more than 50% of vaccine baits disappear within one week and more than 80% are consumed by one to three weeks after distribution^18–22^. Moreover, foxes usually need three nights to search their entire territory for food^23^; therefore, baits should ideally remain stable for at least three days under any weather conditions. A variety of reasons have been proposed to explain variability in bait uptake, including poor bait palatability or availability, variations in the availability of preferred food or monopolisation of baits by some members of a fox population^15,24,25^. Research in Australia focussing on the influence of habitat and fox demographics on poisonous bait uptake (rabies is not occurring in wild foxes in Australia) confirmed that some individuals within a fox population may not remove baits^26,27^, while other foxes may habitually remove and cache baits^28^ in order to reduce the likelihood of bait being discovered by other foxes^29^. As cached baits are not eaten instantly and might be stored for some time this could result in the vaccine virus losing its viability. In addition, evidence for proportionally larger consumption of baits by foxes less than one year old has been found in urban Melbourne^25^. The authors also concluded that baits were either monopolised by young foxes or were less desirable to older foxes. Similar fox behaviour might be possible in Montenegro with home ranges of approximately 3 km^2^ in Australia being comparable to those in Montenegro^30^. Only foxes older than one year were used in the evaluation of the rabies antibody prevalence following the oral vaccination program in Montenegro.

It has been reported that in some cases only the bait matrix is consumed by free-ranging carnivores, but the blister is left behind^31^ and that foxes can eat the bait envelope without piercing the capsule (http://www.fao.org/ag/againfo/programmes/en/empres/gemp/avis/b058-rabies/mod0/0413-4-uptake-sero-con.html). Other research highlighted that poor bait uptake by cats was most likely a result of reduced bait attractiveness or palatability of decaying baits (http://www.terrestrialecosystems.com/cat-baiting-using-eradicat-and-curiosity-is-it-working/). Delayed uptake of bait might result only in a partial consumption of the unattractive bait envelope, thus resulting in positive biomarker results, but not piercing of the vaccine blister and therefore in a failure in developing vaccination titres.

Our results indicate that due to the delayed uptake of bait the rabies vaccine was already inactivated. Various studies demonstrated the negative impact of temperature on the viral titre and on bait matrix integrity^32–36^. Using the vaccine SAD Bern strain (Lysvulpen) Maciulskis *et al*. (2008) showed a virus titre decrease of 2 log in baits exposed to temperatures of 40 °C in the field and highlighted that exposure to rainy weather was detrimental to bait casing stability, making it soft and sticky 24 hours after bait deposition in the field. The same authors also reported differences in bait stability in different landscapes: baits and vaccine titres were stable for nine days in a forest environment and only six days on the edge of the forest^35^. In addition, laboratory based *in vitro* and *in vivo* studies have highlighted that freezing and thawing cycles can impact on vaccine quality^37^. However, to our knowledge evaluations of bait stability have only been conducted in the field for exposure to high temperature and rain, but the impact of snow and lower or freezing temperatures on vaccine virus stability has not been assessed. We calculated the ‘mean temperature until 2 weeks after the vaccination campaign’, but could not find any association with reduced rabies vaccine antibody prevalence. However, temperature values derived from MODIS satellite images might not represent the actual ground temperatures where baits were located.

During the 2011 vaccination campaign in Montenegro, higher fox density per square kilometre was associated with decreased odds of being seropositive. It has been suggested that fox density is determined by the availability of food and suitable habitat^38^. In areas of high fox or fox family density, foxes might not take up the vaccine bait instantly, as other food and prey options are abundant, while in low density areas food and prey might be limited and bait is taken up faster. It has been shown that bait uptake by cats was negatively correlated with the abundance of alternative prey species^39^ and this might be also true for foxes. In addition the habitually removal and caching of baits be more common in higher fox density areas. As the principle of oral vaccination against rabies is for every fox to have access to vaccine baits^40^, enough baits for all animals residing within a territory must be provided^41^. In fact baits must be distributed evenly in order to increase the chance of more individuals having access to bait as opposed to providing more baits to those already reached^9^. The most suitable strategy is therefore the reduction of the flight-line distance^9^. Locations of higher fox density might require more intense monitoring and specific actions during future campaigns. Thus in areas with localised high fox density, surplus or more densely aerial distribution or even hand baiting could be considered a useful approach during future vaccination campaigns. It also has to be considered that although vaccination decreases the number of rabies cases, it can also contribute to a rise in fox density^36,41–43^. In situations of continued vaccination campaigns, it is crucial to compensate for the higher abundance of the vector species through an adjustment of the vaccine bait distribution.

It has been pointed out that spatial landscape heterogeneity influences the spread of rabies between target species^44–46^ and it might also impact on the success of oral vaccinations. Research comparing lethal bait uptake of foxes in different habitats in Australia highlighted that bait uptake was significantly higher in roadside vegetation and along vegetated creek lines than it was in open paddocks^47^. Our modelling has shown that in particular in 2011 increased grasslands cover reduced rabies vaccination antibody prevalence. Grassland areas are usually pastures that are used for grazing or pastures that are harvested mechanically. It is known that foxes tend to move less across larger open areas such as pastures, but rather along areas that provide better protection for them, for example vegetation edges or forest borders and these might be the areas where they most likely pick up bait. On the other hand, increased distance from open spaces with little or no vegetation, which represent bare rock and sparsely vegetated areas in the CORINE LAND COVER data, also reduced rabies vaccination antibody prevalence. These landscape features add to diversity in fox habitats and are therefore attractive environments for foxes where bait is probably easy to spot, while in areas away from these habitat the bait might be more difficult to find. Overall bait might be ‘easier’ to find in good fox habitats, which would have resulted probably in faster bait uptake after aerial distribution. In 2012, higher NDVI values were associated with increased rabies vaccination antibody prevalence. During the spring 2012 vaccination campaign, heavy snowfall was reported^48^ and therefore, lower NDVI values may represent areas covered in snow (where bait might have been difficult to find by foxes) or open grassland areas that as outline above, are often avoided by foxes. However, we explored the variable ‘days of snow cover until 2 weeks after the campaign’ but could not find any association with rabies vaccination antibody prevalence. Lower NDVI values may also represent open grassland areas that, as outlined above, are often avoided by foxes.

The findings of this study should be interpreted in light of a few limitations. First, the number of foxes shot and submitted was moderate and only two successive vaccination campaigns were studied, with high variation in weather conditions between them. Nevertheless, these campaigns were highly successful, highlighted by the rapid decline in rabies incidence in Montenegro. It also has to be considered that Montenegro is a small country, and the decline of rabies incidence was likely influenced by oral vaccination campaigns of foxes conducted in the neighbouring countries of Kosovo, Serbia and Croatia in 2011 and 2012. Second, despite bait density being below the aimed 20 baits per square kilometre in approximately 43% of the national territory of Montenegro^11^, this did not seem to have impacted the odds of foxes developing rabies vaccine antibodies. Third, the quality of serum or body fluids taken from shot foxes may have influenced the detection of rabies vaccination antibodies. However, to address this problem, a ELISA unaffected by the poor quality of serum was developed and is used by several European teams^49^. This ELISA was also applied by the DVL in Montenegro and therefore we are confident that the serological results obtained in this study can be trusted.

Ultimately, the success of an oral vaccination program is evaluated by analysing the incidence of rabies in animals before, during and after the oral vaccination programme. No cases of rabies in foxes have been reported in Montenegro since 2013 (http://www.who-rabies-bulletin.org/Queries/Surveillance.aspx).

## Conclusions

Most research into the direct success of oral vaccination campaigns focussed on the evaluation of bait uptake or only on an estimation of rabies vaccination antibody prevalence. To our knowledge this is the first study that described the impact of environmental, climatic, fox density and bait density factors on rabies vaccination antibody prevalence. Overall we were able to show that rabies vaccination antibody prevalence is impacted by landscape features, with baits in open grassland or snow-covered areas are more likely to result in poor vaccination success, probably due to the delayed uptake of bait. In addition, high fox density as an indicator of good fox habitat might also be associated with delayed bait uptake. Thus delayed bait uptake results most likely in an uptake of inactive rabies vaccine virus.

## Electronic supplementary material

Supporting Information.
