# Supplementary material for: Factors influencing the success of aerial rabies vaccination of foxes
Source: Sci Rep. 2017 Oct 30;7:14376. doi: 10.1038/s41598-017-14615-2 (PMC5662741; doi:10.1038/s41598-017-14615-2)
Supplement: Supplementary file 1 — Supporting Information. [file 41598_2017_14615_MOESM1_ESM.pdf]

Factors influencing the success of aerial rabies vaccination of foxes

J. Henning[1], E. Giorgi[2], R.J. Soares Magalhẽs[1,3], P. Tizzani[4],

P. Viviani[5], N. Pejovic[6], M. Hrapović[7], C. Potzsch[8]

[1] School of Veterinary Science, The University of Queensland, Gatton, 4343, Qld, Australia
[2] Faculty of Health and Medicine, Furness College, Lancaster University, Lancaster, LA1 4YW, United Kingdom
[3] Childrens Health and Environment Program, Child Health Research Centre, The University of Queensland, South Brisbane, 4101, Qld, Australia
[4] Department of Veterinary Sciences, University of Turin, Grugliasco (Torino), 10095, Italy
[5] Veterinary consultant, Perugia, 06123, Italy
[6] Diagnostic Veterinary Laboratory, Podgorica, 81000, Montenegro
[7] Veterinary Administration of the Republic of Montenegro, Podgorica, 81000, Montenegro
[8] Veterinary epidemiology consultant, Tramnitz, 16866, Germany

Corresponding author: : Joerg Henning, j.henning@uq.edu.au

Supporting information

1 Testing residual spatial correlation

Let y_i denote the number of foxes with rabies antibodies out of n_i at location x_i. We define the Person's residuals as

$$\hat{Z}_i = \frac{y_i - n_i \hat{p}_i}{\sqrt{n_i \hat{p}_i (1 - \hat{p}_i)}},$$

where $\hat{p}_i$ is the predicted probability that a fox has rabies antibodies.

We define the empirical semivariogram of the residuals $\hat{Z}_i$ as

$$\hat{V}(u) = \frac{1}{2|N(u)|} \sum_{(h,k) \in N(u)} (\hat{Z}_h - \hat{Z}_k)^2, \tag{1}$$

where $N(u) = \{(i, j) : ||x_i - x_j|| = u\}$, i.e. the set of all pairs of data-points such that their Euclidean distance is u, and $|N(u)|$ is the number of pairs within the set. Intuitively, in the presence of residual spatial correlation in the data, we would expect the squared difference $(\hat{Z}_h - \hat{Z}_k)^2$ to be smaller, on average, at smaller distances u, as a result of the stronger correlation between $\hat{Z}_h$ and $\hat{Z}_k$. Conversely, in the absence of spatial correlation, the empirical semivariogram would only show random fluctuations around a constant value, since $\hat{Z}_h$ and $\hat{Z}_k$ would vary independently of each other at any given distance u.

To reliably distinguish the first case from the latter, we use the following Monte Carlo strategy to simulate empirical semivariograms under the assumption of spatial independence.

1. Permute the locations x_i while holding fix the order of the $\hat{Z}_i$.
2. Compute the empirical semivariogram (1) using the permuted set of locations.
3. Repeat 1 and 2 a large enough number of times, say B.
4. Based on the resulting B empirical semivariograms, compute the 95% confidence intervals for each of the pre-specified distance bins.

If the empirical semivariogram lies outside outside the 95% tolerance bandwidth of step 4, we then interpret this as evidence of residual spatial correlation. If, instead, the empirical semivariogram lies within the 95% bandwidth, we then conclude that the data do not show evidence of residual spatial correlation.

2 Assessing the impact of home ranges above 1 km^2 on parameter estimation and predictions

In this section, we shall use $\tilde{x}$ to denote the location where the fox consumed the bait.

Since $\tilde{x}$ may not coincide with the location x, where the fox was shot, we model $\tilde{x}$ as a inhomogeneous Poisson process (Diggle, 2014) with intensity $\lambda(\tilde{x})$ given by the fox density at location $\tilde{x}$. Let h denote the home range of a red fox, measured in km^2; since each grid cell has an area of 1 km^2, we then consider $h \in \{1, 2, 3, 4\}$ (Cavallini & Lovari, 1994). For a given home range h, we then define all possible combinations with adjacent grid cells to x, such that the total area is h. Figure 1 shows an example for $h = 2$ km^2. In general, we have that the total number of possible habitat configurations, for a given a home range h, is

$$N_h = \binom{8}{h-1} = \frac{8!}{(9-h)!(h-1)!}.$$

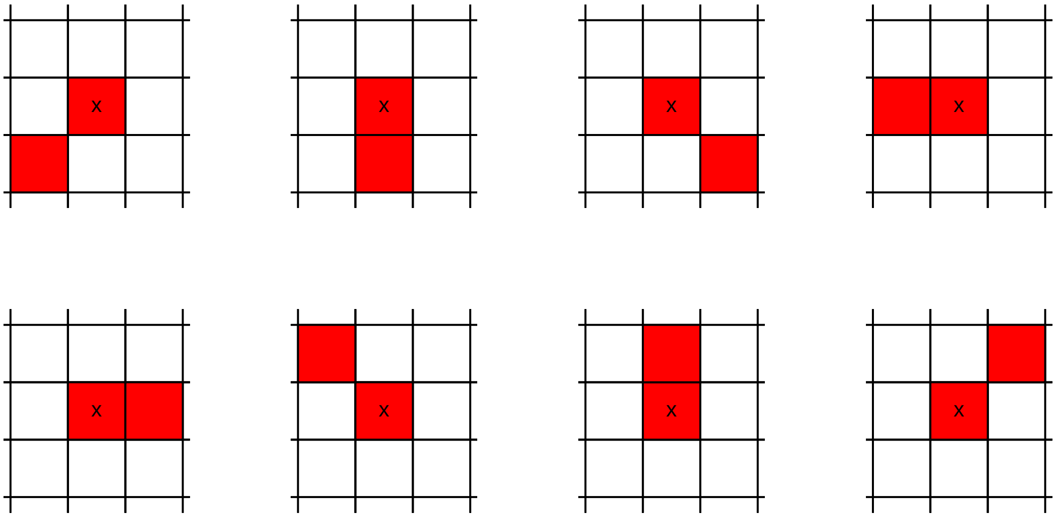

Figure 1: Set of all eight possible habitat configurations for a home range of 2 km^2. Each grid cell has an area of 1 km^2. The central cell, identified by the symbol x, corresponds to the location where the fox was shot.

The log-likelihood function for the regression coefficients β, based on the binary outcome y_i, for $i = 1, \dots, n$, indicating the presence/absence of rabies antibodies is then given by

$$l(\beta) = \sum_{i=1}^{n} \log \left\{ \frac{1}{N_h} \sum_{j=1}^{N_h} \frac{\sum_{\tilde{x} \in \mathcal{C}_j} \lambda(\tilde{x}) e^{l_i(\beta|\tilde{x})}}{\sum_{\tilde{x} \in \mathcal{C}_j} \lambda(\tilde{x})} \right\}, \tag{2}$$

where $\mathcal{C}_j$ is the j-th habitat configuration out of N_h, and

$$l_i(\beta|\tilde{x}) = y_i \eta(\tilde{x}) - \log \left(1 + e^{\eta(\tilde{x})}\right), i = 1, \dots, n$$

with linear predictor $\eta(\tilde{x}) = d(\tilde{x})^\top \beta$ and explanatory variables $d(\tilde{x})$. To estimate β, we then maximize (2) using numerical optimization. Standard errors are computed by taking the square root of the diagonal elements of the inverse of the negative Hessian matrix at the maximum likelihood estimate.

Figure 2 and Figure 3 show the point estimates with associated 95% confidence intervals for 2011 and 2012, respectively. Table 1 and Table 2 report the percentage relative change in the estimates of the regression coefficients with respect to those at a home range of 1 km^2, for 2011 and 2012, respectively. For example, standardized percentage of grass has a relative change of 10.68% at a home range of 4 km^2 with respect to a home range of 1 km^2.

Figures 4 and 5 report the predictions for the probability of presence of rabies antibodies and their associated standard errors, for the four different levels of home range. In the 2011, the changes are intangible for the both the point predictions and standard errors, whilst for 2012, we observe a slight increase in the standard errors as home range increases.

References

Cavallini, P. & Lovari, S. (1994). Home range, habitat selection and activity of the red fox in a Mediterranean coastal ecotone. *Acta Theriologica* **39**, 279–287.

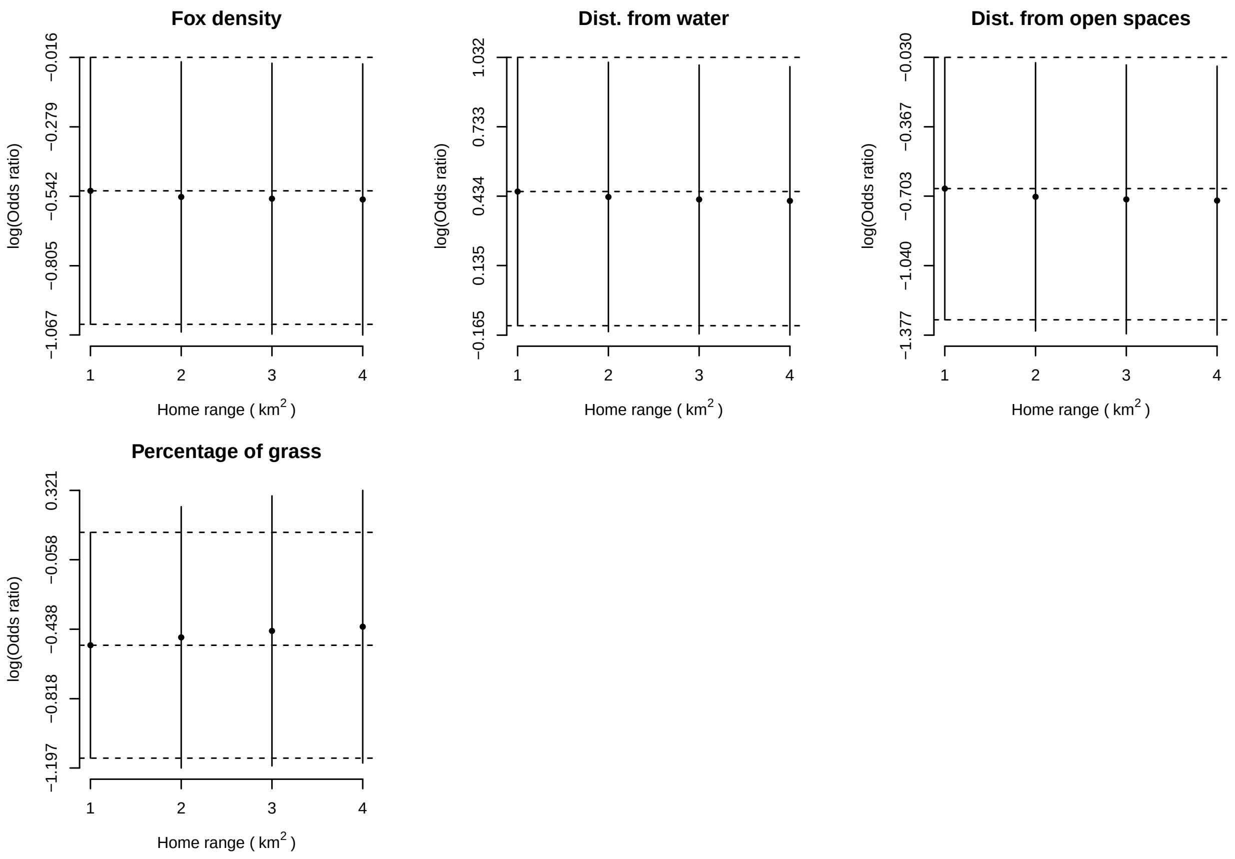

Figure 2: Year 2011: Point estimates and 95% confidence intervals for the log-transformed odds ratios for each of the explanatory variabes, including the interecept, against the different values of home range. The dashed horizontal lines are used as a reference for the values at a home range of 1 km^2.

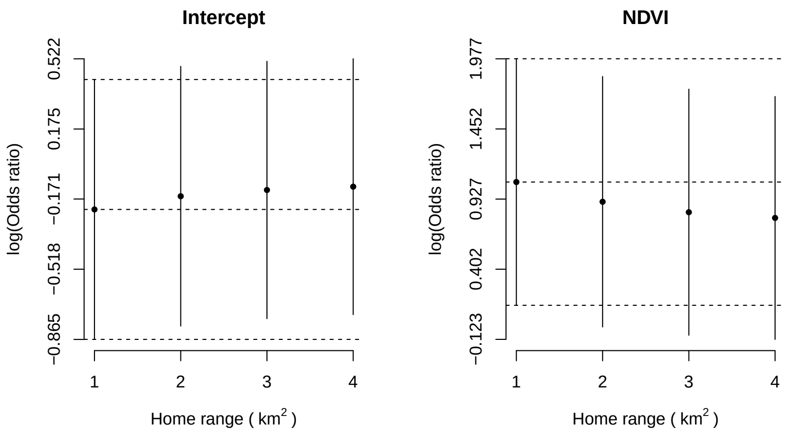

Figure 3: Year 2012: Point estimates and 95% confidence intervals for the log-transformed odds ratios for each of the explanatory variabes, including the interecept, against the different values of home range. The dashed horizontal lines are used as a reference for the values at a home range of 1 km^2.

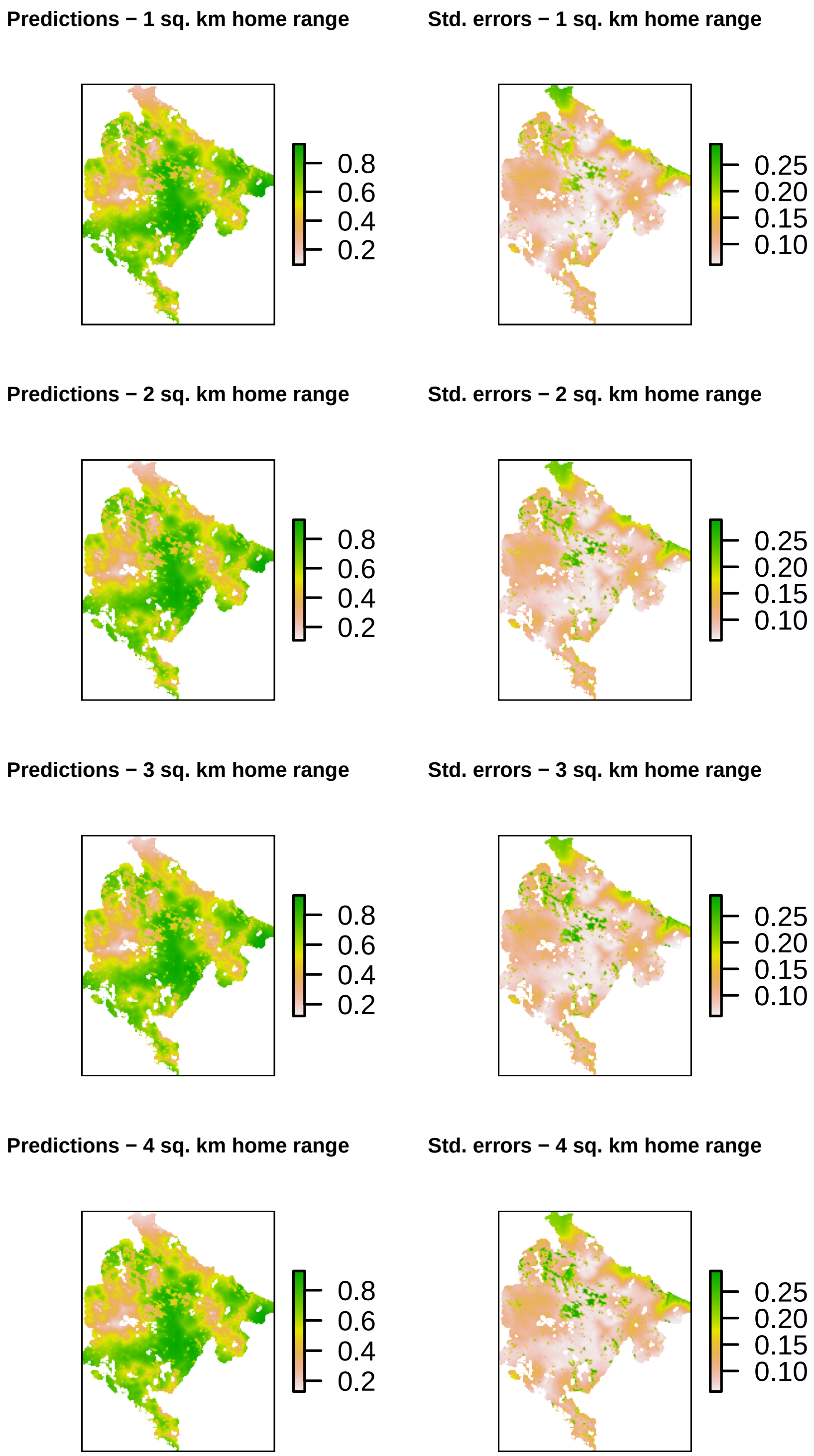

Figure 4: Predictions for 2011: predictions of the probability of presence of rabies antibodies (left panels) and standard errors (right panels). Each row corresponds to a specific home range as indicated by the title of each panel. The maps were generated using the R software environment (R Core Team, 2012), version 3.3.1 (`http://cran.r-project.org`).

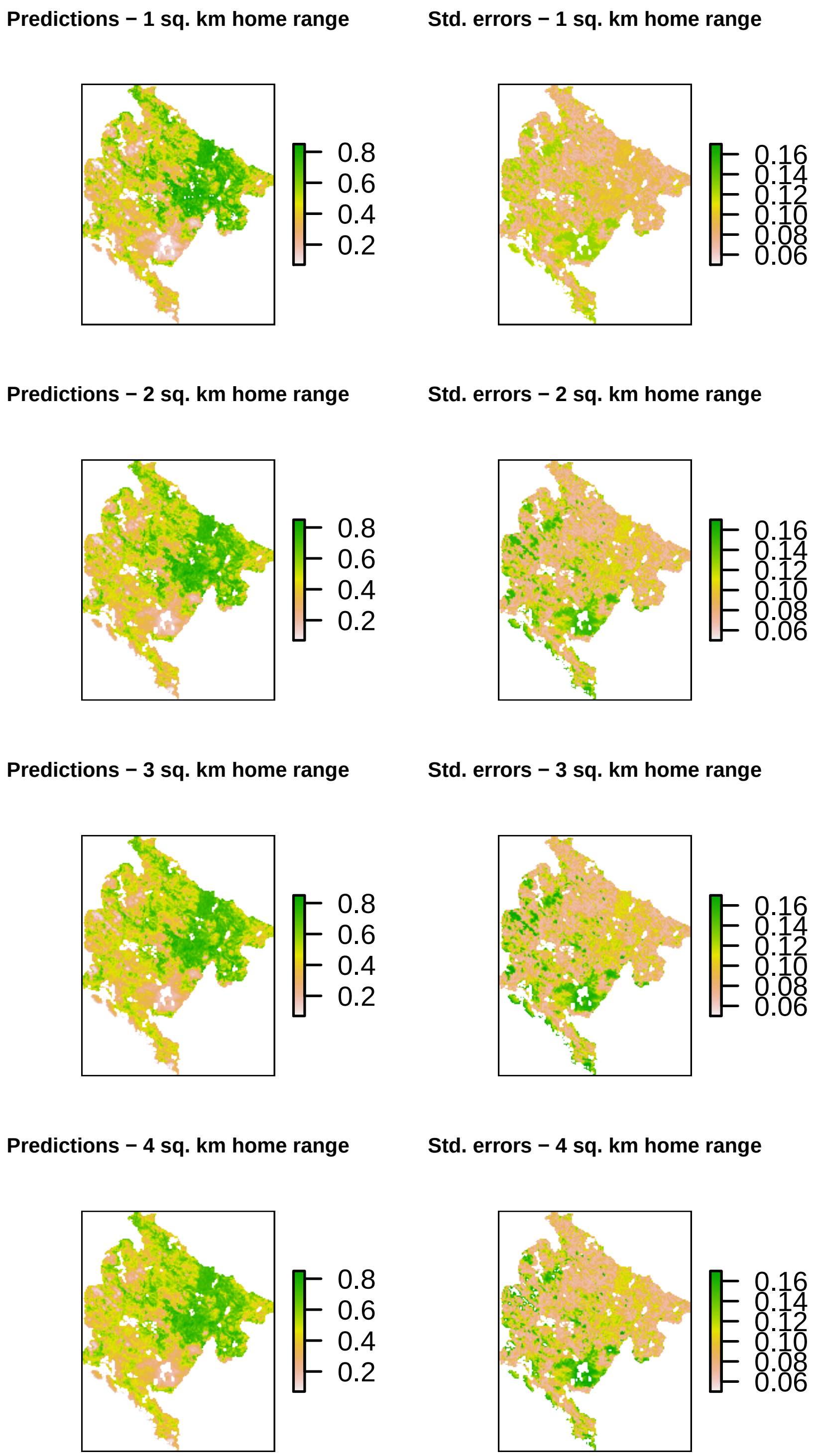

Figure 5: Predictions for 2012: predictions of the probability of presence of rabies antibodies (left panels) and standard errors (right panels). Each row corresponds to a specific home range as indicated by the title of each panel. The maps were generated using the R software environment (R Core Team, 2012), version 3.3.1 (`http://cran.r-project.org`).

Diggle, P. J. (2014). *Statistical Analysis of Spatial and Spatio-Temporal Point Patterns*. CRC/Chapman & Hall, Boca Raton.

R Core Team (2012). *R: A Language and Environment for Statistical Computing*. R Foundation for Statistical Computing, Vienna, Austria. ISBN 3-900051-07-0.

Table 1: Year 2011: Percentage relative difference in the point estimates of the regression coefficients with respect to a home range of $1km^2$.

Home range (km^2)	Intercept	Fox density	Dist. from water	Dist. from open spaces	Percentage of grass
2	0.34	2.28	2.31	3.91	4.41
3	0.14	2.93	3.36	5.11	8.16
4	0.05	3.22	3.95	5.66	10.68

Table 2: Year 2012: Percentage relative difference in the point estimates of the regression coefficients with respect to a home range of $1km^2$.

Home range (km^2)	Intercept	NDVI
2	6.74	13.73
3	10.09	20.23
4	11.92	23.57
